# Childhood to adult transition in youth patients with lysosomal acid lipase deficiency: 43 recommendations from experts

**DOI:** 10.1186/s13023-025-03852-8

**Published:** 2025-07-02

**Authors:** Alvaro Hermida-Ameijeiras, Javier Blasco-Alonso, Juan Luis Carrillo-Linares, María Luisa González-Dieguez, José Pastor-Rosado, Montserrat Morales-Conejo, Marcello Bellusci, Maria Mercadal-Hally

**Affiliations:** 1 Division of Internal Medicine, University of Santiago de Compostela. UETeM- Molecular Pathology Group, IDIS-CIMUS, MetabERN, Santiago de Compostela, Spain; 2 Section of Pediatric Gastroenterology and Nutrition, Regional University Hospital of Malaga, Malaga, Spain; 3 IBIMA Multidisciplinary Pediatric Group, Malaga, Spain; 4 Internal Medicine Department, Virgen de la Victoria University Hospital, Malaga, Spain; 5 Liver Unit, Division of Gastroenterology and Hepatology, Central University Hospital of Asturias, Oviedo, Spain; 6 Lipid Unit, Department of Pediatrics, General University Hospital of Elche, Miguel Hernandez University of Elche, Elche, Spain; 7 Internal Medicine Department, Biomedical Research Center CIBERER, CSUR for Congenital Metabolic Disorders, Octubre University Hospital, Madrid, U723 Spain; 8 Reference Center for Inherited Metabolic Disorders, MetabERN Center, 12 de Octubre University Hospital, Madrid, Spain; 9 Pediatric Hepatology and liver transplant Unit, Vall d’Hebron Hospital, Barcelona, Spain

**Keywords:** Adolescents, Lysosomal acid lipase deficiency, Multidisciplinary consensus, Pediatric to adult care transition, Recommendations, Transition protocol

## Abstract

**Background:**

The process of transition from pediatric to adult care is crucial, especially in rare diseases such as lysosomal acid lipase deficiency (LAL-D). Unfortunately, this process is associated with poor outcomes, and many challenges still await to be addressed. This document provides recommendations on the pediatric to adult care transition in patients with LAL-D, based on available evidence and the experience of a panel of experts, which include specialists in the management of patients with LAL-D, and representative patients of the AELALD patient organization. Additionally, the main uncertainties and/or challenges encountered by the different stakeholders during the process are defined.

**Main body:**

A total of 43 consensus recommendations were developed across 5 areas. The consensus recommendations reflect the personal opinions and experiences of the participating experts supported with evidence when available. Overall, the main uncertainties and/or challenges faced comprise the patient’s mistrust in the new medical team, the insufficient information received, or the lack of time, resources and institutional support. The management of adolescents/young adults during the transition to adulthood should be a joint effort between the patient, clinical center, and parents/caregivers. The objective of the transition process should be to empower patients and progressively encourage the self-management of their disease, and therefore patients and their families should be involved in all phases of the transition. Facilitating elements, such as standardized protocols, arose as important tools to ease the transition process.

**Conclusions:**

This multidisciplinary consensus provides information on the main obstacles found by patients and their families, pediatricians and adult physicians during the transition from pediatric to adult care in LAL-D. To overcome these barriers, the scientific committee highlights the need for different facilitating elements, which include an effective collaboration between healthcare teams, the elaboration of standardized protocols, the implementation of education for patients, or the individual evaluation of the need for psychological support. The recommendations provided by the experts are the basis to facilitate and improve the transition process.

**Supplementary Information:**

The online version contains supplementary material available at 10.1186/s13023-025-03852-8.

## Background

Lysosomal acid lipase deficiency (LAL-D) is a rare autosomal recessive disease with an estimated prevalence of 1 in 250.000 in children and adults [1]. LAL-D patients present with variable clinical manifestations, including hepatic steatosis and liver failure, atherosclerosis, cardiovascular disease and premature death [2]. Due to its similarity to other metabolic diseases, LAL-D diagnosis is challenging [2], and the disease is often underdiagnosed [3]. This, together with the fact that LAL-D is multifactorial, and patients are monitored by many different specialists [4, 5], strengthens the need for protocols and guidelines for the correct management and monitoring of LAL-D patients [4, 5].

The transition of patients from pediatric to adult care is of great importance in chronic diseases, especially in rare diseases such as LAL-D [3]. However, the moment of transfer to adult care is unfortunately associated with missed medical consultations, reduced adherence to treatments and therefore poor outcomes [6]. Moreover, the poor sanitary organization and lack of information and resources make this process very complex and with many challenges ahead [7].

Although there are some guidelines with recommendations about the management of specific diseases and the main goals and difficulties found during a transition process, they are mainly established locally and cannot be implemented on a wider level [8] and there is no consensus directed specifically to LAL-D. The aim of this consensus is to provide recommendations on the pediatric to adult care transition of LAL-D patients based on available evidence and expert clinical judgement. Additionally, the main uncertainties and/or challenges encountered by the different specialists at the moment of transition from pediatric to adult care are described.

## Methods

The current document is the result of a systematic literature review and a qualitative investigation with a scientific committee, which comprised different specialists with extensive experience in the management of patients with LAL-D and LAL-D patients themselves, representatives of the AELALD patient organization (Spanish Association for LAL-D). All participants came from different healthcare settings and from different parts of Spain.

Twelve clinical questions using the PICO format (Patient, Intervention, Comparison, Outcomes) [9] and grouped in 5 relevant topics were elaborated. Relevant topics and questions are included in Table 1.

**Table 1:** List of relevant topics and questions to be answered

Topic 1: Uncertainties and challenges	
Question 1	What are the main uncertainties and/or challenges for patients with LAL-D and their families during the transition process from adolescence to adulthood?
Question 2	What are the main uncertainties and/or challenges for pediatricians of patients with LAL-D during the transition process from adolescence to adulthood?
Question 3	What are the main uncertainties and/or challenges for adult physicians of patients with LAL-D during the transition process from adolescence to adulthood?
Topic 2: Facilitating elements	
Question 4	What are the main facilitating elements for the transition process of a patient with LAL-D?
Topic 3: Transition	
Question 5	What clinical and care information is important to convey to the patient and/or family members about LAL-D throughout the entire transition process?
Question 6	What competencies must they have achieved at the time of transition or are recommended to be achieved at the time of transition and what responsibilities should they acquire during the transition process?
Question 7	What situations might hinder or restrict the transition process?
Question 8	What should be included in the transition plan of a patient with LAL-D? (information for patients, primary care and adult physician).
Topic 4: Adherence, self-care and monitoring	
Question 9	What measures or methods are useful to improve therapeutic adherence of patients with LAL-D during the transition process?
Question 10	What measures or methods are useful to favor self-care and involvement of adolescent and young adult patients with LAL-D during the transition process?
Topic 5: Education and training	
Question 11	What initiatives related to education and training of the entire medical community are recommended for the transition of patients with LAL-D?
Question 12	What initiatives related to education and training of patients and their families are recommended for the transition of patients with LAL-D?

LAL-D: lysosomal acid lipase deficiency

### Systematic literature review

To conduct the literature review, a systematic *rapid review* was carried out using the methodology of mixed-methods systematic review (MMSR) [10]. Publications related to LAL-D from the following databases were included: National Guideline Clearinghouse, National Library of Guidelines, TRIP database, Epistemonikos and MEDLINE Cochrane Database of Systematic Reviews (CDSR), PubMed, EMBASE and Database of Abstracts of Reviews of Effects (DARE). For the search for primary studies and grey literature, the Google search engine was also used. The strategy employed during the search is shown in Table S1. A stepwise search was conducted, and two review authors independently assessed articles according to inclusion criteria by title and abstract. Any discrepancies were resolved through discussion or consultation with a third reviewer. The Rayyan QCRI^®^ was used as data management software for the two screening phases. The assessment of the risk of bias of the included studies was carried out using the indicated tool depending on the design of each study (e.g. RoB, Robins or AMSTAR-2). An evaluation of the quality and the assessment of the evidence was carried out using the Scottish Intercollegiate Guidelines Network (SIGN) approach [11].

### Qualitative investigation

Three virtual Focus Group were conducted to identify the current needs and challenges associated with the pediatric to adult care transition of LAL-D patients. Each Focus Group contained five or six participants and was represented by (i) specialists involved in the management of children with LAL-D, (ii) specialists involved in the management of adults with LAL-D, (iii) LAL-D patients and their families. The content of the virtual meetings was developed by a steering committee of two experts who also led the meetings. The Focus Group served as a critical platform for capturing experience-based insights from both clinical and patient perspectives, providing an understanding of transition-related gaps and priorities.

### Elaboration of the recommendations

The evidence gathered in the three Focus Group and the information from the systematic literature review were analysed to answer and contextualize the clinical questions and to elaborate recommendations.

The percentages of agreement and disagreement were calculated using the Likert scale. Consensus was considered as agreement ≥ 80% [12]. An agreement of 100% was considered unanimity. Discrepancy was considered when the agreement was found between 66% and 79%. Rejection was considered when the agreement was lower than 66%.

### Main body

We included a total of 37 publications, including 10 clinical practice guidelines, 12 systematic reviews and 15 other studies. Initially, we identified 35 studies susceptible to be included. From these, 20 were discarded because of: (i) target pathology is not correct (10 publications), (ii) articles do not answer any question (7 publications), and (iii) the study type (3 publications). Table S2 shows the characteristics of the different publications included.

### Consensus process results

#### Topic 1: uncertainties and challenges

The main uncertainties and/or challenges encountered by patients with LAL-D and their families, pediatricians and adult physicians were defined. For patients, the main gaps comprised the patient’s mistrust in the new medical team, insufficient information received concerning LAL-D or self-care and difficulties in future planning due to the differences in the structure and management of pediatric and adult hospital services. Pediatricians and adult physicians miss joint communication and reported difficulties in knowing the bureaucratic procedure in referrals, especially between autonomous communities or reference units, which delays or omits patient’s visits.

#### Topics 2–5

The recommendations were updated or modified based on new evidence or the expert judgment of the participants.

Table 2 shows the final recommendations and the percentage of agreement for all the proposed topics.

**Table 2:** Final recommendations and % of agreement

Question	Recommendation	% agreement
	Topic 2: Facilitating elements	
4	1. To ensure a successful transition process, sufficient time must be allocated, and an efficient organizational structure should be established. This structure should include a multidisciplinary team (metabolic specialists, hepatologists, nutritionists, psychologists, and social workers) familiar with LAL-D and its specific needs, such as enzyme replacement therapy, ongoing hepatic and cardiovascular monitoring, and tailored dietary support.	100%
	2. The active involvement of a transition manager with expertise in rare metabolic diseases is strongly recommended. This professional can play a key role in coordinating and facilitating the transition from pediatric to adult care, ensuring a comprehensive approach.	83%
	3. There must be communication between the healthcare teams: ‒ Shared medical records can facilitate communication. ‒ New technologies (such as videocalls) allow communication between the different professionals overcoming physical barriers (different healthcare centres, day hospitals…).	100%
	4. The involvement of the entire healthcare team is needed so that they work in a collaborative, coordinated manner and create protocols for this.	100%
	5. A previous contact between the adolescent with LAL-D and the adult physicians before the transition or joint medical consultations with the pediatrician and the adult physician are recommended.	100%
	6. The specific transfer moment to adult care must be based on the appropriate time for the patient and it should not be determined by a specific age.	83%
	7. Training and education of LAL-D patients about their disease are crucial. The important role of patient associations must be taken into account.	100%
	8. Patients should be provided with information about the disease, including its genetic basis, clinical progression, available treatment options (such as enzyme replacement therapy with sebelipase alfa), and the importance of long-term follow-up, particularly regarding liver and cardiovascular health. They should also receive relevant information related to adult services and patient associations (contact numbers, location, hours, etc.).	100%
	9. It is recommended to use facilitating tools, such as competency checklists, to plan and correctly monitor the transition, as well as to implement video consultations to improve and expedite the process.	100%
	10. The need of psychological support during the process of transition should be evaluated individually.	83%
	11. It is recommended to write a complete medical record including all clinical and psychosocial aspects during the transition process.	67%
	12. An active role of patient organizations and families in the transition process is encouraged (through distinct mechanisms such as meetings and forums with joint participation of families and professionals, elaboration of documents…).	83%
	13. The role of the family doctor must be highlighted as a facilitating element, and therefore, they must be involved in the process by providing them with relevant clinical information and means of contact with other professionals.	83%
	Topic 3: Transition	
5	14. Patients should be informed about the nature of the disease including its genetic origin. It is recommended to provide written information and use language adapted for correct interpretation.	100%
	15. Patients and families should be educated on the importance of enzyme replacement therapy (ERT) with sebelipase alfa, including dosing intervals, potential infusion-related reactions, and the irreversibility of liver damage if left untreated. This is key to long-term treatment success.	100%
	16. It is recommended that patients receive information about the progression of the disease, including cardiovascular risk. It is advised that they receive detailed information about healthy lifestyle guidelines (diet, exercise, avoiding toxins), sexually transmitted diseases, pregnancy, genetic counselling, as well as the resources they should access periodically.	100%
6	17. The transition approach towards self-management of a chronic illness in which the responsibility is assumed progressively, starting in the pediatric setting and consolidating during the transition process in the adult service is recommended.	100%
	18. Patients must have acquired knowledge about their disease and develop skills to get on in the adult consultation, which involves understanding the requested tests and actively participating in follow-up consultations.	100%
	19. The creation of a list of competencies (“check-list”), which facilitates the acquisition of skills, and its monitoring is recommended.	100%
	20. Patients with LAL-D must have acquired knowledge that allows them to understand the nature of their disease and the complications that may occur.	100%
	21. It is advised that patients acquire knowledge about their treatment (enzyme replacement therapy and the importance of adherence to treatment schedules) and prevention of risks associated with LAL-D (e.g. cardiovascular diseases), such as lifestyle guidance, including dietary recommendations tailored to LAL-D and the avoidance of hepatotoxic substances.	100%
	22. It is recommended that patients develop skills to manage well in the adult unit, understanding the follow-up that is required (hepatologist or experts in lipids who will carry out their follow-up, tests that are requested, management of appointments, required medication…).	100%
7	Information indicated in Topic 1: Uncertainties and challenges	-
8	23. The transition plan must be protocolized and presented physically or digitally. It should be customizable and adapted to the patient’s language. It is important that it includes the patient and family’s contact information, as well as information about the psycho-social situation.	100%
	24. The transition plan must include the healthcare circuits for patients with LAL-D, such as unscheduled urgent care or the functioning of the day hospital for the administration of treatment.	100%
	25. It is recommended to protocolize the transition plan in phases with different objectives: a. Phase 1: “Know what is happening to me”. b. Phase 2: “Know how to take care of it”. c. Phase 3: “Consolidation phase”.	100%
	Topic 4: Adherence, self-care and monitoring	
9	26. The transition process must identify low adherence risk and apply mechanisms to improve it.	83%
	27. Diet plan should be reviewed during the transition process both with the patient and family. Patients and caregivers should receive individualized dietary counselling led by a metabolic dietitian, emphasizing a low-cholesterol, low-saturated fat diet, without over-restriction that could impact growth and development.	83%
	28. Monitoring should include both pharmacological adherence (e.g., to sebelipase alfa) and nutritional compliance, as well as review past complications and current schedule. Strategies like medication diaries, mobile apps, telehealth reminders, and family-based coaching may help reduce non-adherence in young patients.	100%
10	29. The provision of information, with periodic updates, should be considered to ensure the understanding of the patient and their family/caregivers.	83%
	30. Having tools such as the list of competencies (“checklist”) or roadmap will allow greater involvement of the patient and objectify their evolution during the process.	100%
	31. Convey the importance of self-care, involvement with the disease and healthy lifestyle habits (avoid toxics consumption and encourage physical exercise). It should be carried out with the participation of the family from adolescence along with periodic reinforcement from health professionals.	100%
	32. A nutrition-focused transition plan should be developed, including baseline and annual lipid profiles and liver enzyme monitoring, counselling on nutrient-dense alternatives in restricted diets and screening for vitamin D deficiency and bone health in case of restricted fat intake.	100%
	33. Direct contact with adolescents should be encouraged and the use of telecare tools should be valued.	100%
	34. It is recommended to connect adolescents with lysosomal acid lipase deficiency (LAL-D) to patient organizations, since these can play a prominent role in their empowerment during the transition stage.	83%
	Topic 5: Education and training	
11	35. It is recommended to increase education and training opportunities for pediatric clinicians and adult physicians on topics related to transition, adolescent development, and clinical aspects of LAL-D.	100%
	36. Training with clinical cases with the key points on the usual management (pediatric and adult) of patients with LAL-D should be carried out, and collaborative meetings between pediatricians and adult physicians to address specific cases during the time of transition should be organized.	100%
	37. It is recommended that the transition report includes, in addition to the evolutionary summary of the patient’s illness, detailed information for the primary care physician that includes: pharmacological interactions of the patient’s treatment, management of common diseases and contact with the patient’s reference team.	100%
	38. It is recommended that the transition manager contacts the primary care team to send them the aforementioned report and maintains an effective collaboration between teams.	100%
12	39. It is recommended to incorporate training initiatives into the transition plan of each patient with LAL-D.	100%
	40. It is advisable to foster active collaborations with patient organizations since they play a very relevant role in the education and training of patients.	100%
	41. It is recommended to encourage “peer-to-peer” meetings to increase knowledge of the disease, self-care capacity, and autonomy among patients in the process of transition.	100%
	42. The use of various learning strategies such as role-playing, one-to-one interactions, coaching, graphic and visual materials, online resources, and continuous and supervised support should be valued.	83%
	43. It is recommended to actively disseminate through social networks relevant information about LAL-D.	83%

ERT: enzyme replacement therapy, LAL-D: lysosomal acid lipase deficiency

From the 41 recommendations proposed, 40 had an agreement of ≥ 80%, and thus were considered consensus (11 recommendations, agreement ≥ 80%) or unanimity (29 recommendations, 100% agreement). One recommendation included in *Facilitating elements* had an agreement of 67% and was considered a discrepancy.

## Discussion

The process of transition from pediatric to adult care is of great importance, especially in patients with chronic and rare diseases such as LAL-D. According to a systematic review [6], several studies provided evidence concerning the main challenges encountered during the transition process in different pathologies and the steps needed to fill this gap. However, these steps have been mostly implemented at local level and for specific pathologies and cannot be generalized to other diseases and healthcare settings [8].

The need for transition protocols is emphasized in several studies [13, 14], including studies with chronic conditions [15]. A protocolized and structured transition process has been shown to be beneficial to improve the adherence to care, the self-management of the disease and the quality of life of patients [14].

### Multidisciplinary consensus

This consensus provides recommendations on the pediatric to adult care transition in patients diagnosed with LAL-D based on the experience of a panel of different experts and the available evidence. The experts comprised not only pediatricians and adult physicians, but also representative patients of the AELALD patient organization to consider their opinion. It identifies the main challenges faced by pediatricians, adult physicians and the patients and their families during the process of transition; and the points which are important to help coordinate and manage this process.

### Information for patients

This consensus highlights the importance of receiving information about the disease, including its genetic basis, clinical progression, available treatment options (such as enzyme replacement therapy), and long-term follow-up specially regarding liver and cardiovascular health. In line with this consensus, several studies highlighted the importance for the patient to receive information, either about the transition process or the pathology [6–8, 13, 16, 17]. In the German study TRANSLATE NAMSE [8], which included 10 different hospitals in Germany, most of patients presented a knowledge gap at the beginning of the transition process. This study underlined the importance of empowering the patient during the transition process by using individualized training and counselling. The transition to adult care in lysosomal storage diseases was evaluated in the TENALYS study in France [7] aimed at identifying the gaps and actions to improve the transition process through questionnaires answered by patients or their families and pediatricians. This was an observational, non-interventional and cross-sectional national survey and included 7 pediatric investigation centers, all referral centers for inherited metabolic diseases. According to this study, most patients revealed receiving information on the transition process, although in half of them the information was received after they reached adulthood, underlying the need for a planned, standardized and personalized transition process.

### Education and training for physicians

In our consensus, experts recommend increasing the education and training opportunities for pediatric and adult physicians on topics related to LAL-D and the transition process. The scientific committee also recommends disseminating information about LAL-D through social networks. Due to the recent advances in medicine, patients with rare lysosomal disorders survive longer, but adult physicians are not yet familiarized with these diseases [7, 18]. Therefore, physicians often lack knowledge on topics related to rare diseases. The lack of physicians training has been reported as a barrier to face during the transition process [13].

### Patients’ self-management of the disease

Given that the transition process is associated with lower adherence to treatments [6], therapeutic education for patients with LAL-D transitioning to adult care, both in terms of pathology and the importance of the adherence to treatment schedules (e.g. sebelipase alfa), is critical. This consensus highlights the importance of identifying the risks of low adherence and applying mechanisms to improve it, monitoring the intake of lipid-lowering medication at home and providing information with regular updates. Monitoring should also include a review of past complications and current schedule, implementing strategies to reduce non-adherence in young patients [5, 18].

As in this consensus, to achieve the success of the transition, the figure of a transition manager or transition coordinator [7] and the importance of institutional support [14] have been described.

As recommended in guidelines [19], experts from this consensus emphasize that the transition process should be progressive and directed towards the patient’s self-management of the disease. Therefore, the patients should be involved during all the transition process, and tools such as the list of competencies (“checklist”) or roadmaps, the use of telecare and strategies to foster collaborations with patient organizations are encouraged. The proposed checklist can be found in Table S3.

A significant part of self-care for patients with LAL-D is nutritional management. According to a recent guideline [20], this is even a critical aspect during the transition. In this regard, the experts of this consensus also recommend reviewing the dietary plan with a metabolic dietitian during the transition, emphasizing a restricted fat diet [1, 2] and the importance of developing a nutrition-focused transition plan [21], including baseline and annual lipid profiles and liver enzyme monitoring, as well as counselling.

Guidelines strongly recommend performing a multidisciplinary transition process including figures such as a psychologist to consider the psychological distress that patients undergo during the transition process [19]. This consensus contemplates the individual evaluation of the need for psychological support.

Other uncertainties and/or challenges, which are not reported in this study, such as the sociodemographic and cultural status of the patient or developmental immaturity, have been described for other diseases [6].

### Communication between physicians

Different studies and guidelines highlight the importance of effective communication between pediatricians and adult physicians, for example through multidisciplinary consultations [6, 7, 19].

The lack of appropriate medical records has also been reported as a key barrier during the transition process [13]. However, in this study the experts disagreed (67% of agreement) on writing a complete medical record during the transition process. Instead, the scientific committee agreed that physicians should elaborate and share a transition report, which includes detailed information about aspects such as pharmacological interactions of the patient’s treatment or psychosocial issues.

### Facilitating elements

To overcome all the challenges and/or uncertainties, the scientific committee highlights the need for different facilitating elements to favor the transition process for patients with LAL-D. This comprises allocating sufficient time and counting on an efficient organizational structure, including metabolic specialists, hepatologists, nutritionists, psychologists and social workers, in order to ensure a successful transition process. Additionally, it includes institutional support, effective communication, collaboration and coordination between healthcare teams, the elaboration of standardized protocols, the implementation of training and education for patients and the figure of the transition manager. Other studies highlight the need of other facilitating elements, such as the regular assessment of transition readiness [22].

### Study limitations

To our knowledge, this is the first consensus aimed at providing recommendations on the transition from pediatric to adult care specifically directed to LAL-D. The main limitation is the lack of evidence of specific challenges in LAL-D. We ought to salvage this by including not only medical specialists but also patients with LAL-D to include their specific point of view. Nevertheless, many of the recommendations could probably be extrapolated to other rare diseases. It is necessary to conduct further studies to disclose differences between pathologies that may require specific management during the transition to adult care.

The scientific committee participating in this consensus comprised professionals from different parts of Spain. Therefore, the conclusions of this study cannot be extrapolated to other countries or pathologies. Additionally, there may arise difficulties in the implementation of the recommendations in the different medical centers which manage patients with LAL-D due to the heterogeneity in the availability of resources.

## Conclusions

This multidisciplinary consensus allows the identification of the main uncertainties and challenges that patients, pediatricians, and adult physicians face in the process of transitioning from pediatric to adult care in LAL-D. Additionally, it provides a list of recommendations to consider facilitating the transition process.

The implementation of a transition protocol agreed upon by patients and their families, pediatricians and adult specialists will ensure patient adherence to treatment, improve their quality of life and optimize the healthcare system’s care process.

## Electronic supplementary material

Below is the link to the electronic supplementary material.

Supplementary Material 1

Supplementary Material 2

Supplementary Material 3

## Data Availability

Data sharing is not applicable to this article as no datasets were generated or analysed during the current study.

## Abbreviations

AELALD: Spanish Association for LAL-D (Asociación Española de LAL-D)
CDSR: Cochrane Database of Systematic Reviews
DARE: Database of Abstracts of Reviews of Effects
ERT: Enzyme replacement therapy
LAL-D: Lysosomal acid lipase deficiency
MMSR: Mixed-methods systematic review
PICO: Patient, Intervention, Comparison, Outcomes
SIGN: Scottish Intercollegiate Guidelines Network

## References

[1] Burton BK, Silliman N, Marulkar S. Progression of liver disease in children and adults with lysosomal acid lipase deficiency. Curr Med Res Opin. 2017;33(7):1211–4. 28320214 10.1080/03007995.2017.1309371

[2] Reiner Z, Guardamagna O, Nair D, Soran H, Hovingh K, Bertolini S, et al. Lysosomal acid lipase deficiency–an under-recognized cause of dyslipidaemia and liver dysfunction. Atherosclerosis. 2014;235(1):21–30. 24792990 10.1016/j.atherosclerosis.2014.04.003

[3] Marín-Andrés M, Ros-Arnal I, Cebolla-Sanz JJ, Pérez-Delgado R, García-Jiménez MC. [Lysosomal acid lipase deficiency: A rarely recognised cause of dyslipidaemia and liver dysfunction]. Pediatr (Engl Ed). 2021;94(1):50–1. 10.1016/j.anpedi.2020.02.006 32736925

[4] Pérez-López J, Ceberio-Hualde L, García-Morillo JS, Grau-Junyent JM, Hermida-Ameijeiras A, López-Rodríguez M, et al. [Transition process from paediatric to adult care in patients with inborn errors of metabolism. Consensus Statement] Med Clin (Barc). 2016;147(11):506.e1-e7. 10.1016/j.medcli.2016.09.018 27816186

[5] Kohli R, Ratziu V, Fiel MI, Waldmann E, Wilson DP, Balwani M. Initial assessment and ongoing monitoring of lysosomal acid lipase deficiency in children and adults: consensus recommendations from an international collaborative working group. Mol Genet Metab. 2020;129(2):59–66. 31767214 10.1016/j.ymgme.2019.11.004

[6] Gray WN, Schaefer MR, Resmini-Rawlinson A, Wagoner ST. Barriers to transition from pediatric to adult care: A systematic review. J Pediatr Psychol. 2018;43(5):488–502. 29190360 10.1093/jpepsy/jsx142

[7] Genevaz D, Arnoux A, Marcel C, Brassier A, Pichard S, Feillet F, et al. Transition from child to adult health care for patients with lysosomal storage diseases in france: current status and priorities-the TENALYS study, a patient perspective survey. Orphanet J Rare Dis. 2022;17(1):68. 35189927 10.1186/s13023-022-02232-w PMC8862388

[8] Grasemann C, Höppner J, Burgard P, Schündeln MM, Matar N, Müller G, et al. Transition for adolescents with a rare disease: results of a nationwide German project. Orphanet J Rare Dis. 2023;18:93. 37098531 10.1186/s13023-023-02698-2 PMC10131406

[9] Richardson WS, Wilson MC, Nishikawa J, Hayward RS. The well-built clinical question: a key to evidence-based decisions. ACP J Club. 1995;123(3):A12–3. 7582737 10.7326/ACPJC-1995-123-3-A12

[10] Aromataris E, Munn Z, editors. JBI Manual for Evidence Synthesis. JBI, 2020. Available from https://synthesismanual.jbi.global. 10.46658/JBIMES-20-01

[11] Scottish Intercollegiate Guidelines Network (SIGN) A guideline developer’s handbook Edinburgh: SIGN; 2019 (SIGN publication no 50) [November 2019] Available from: http://www.signacuk.

[12] Fink A, Kosecoff J, Chassin M, Brook RH. Consensus methods: characteristics and guidelines for use. Am J Public Health. 1984;74(9):979–83. 6380323 10.2105/ajph.74.9.979 PMC1651783

[13] Szalda DE, Jimenez ME, Long JE, Ni A, Shea JA, Jan S. Healthcare system supports for young adult patients with pediatric onset chronic conditions: A qualitative study. J Pediatr Nurs. 2015;30(1):126–32. 25450439 10.1016/j.pedn.2014.09.015 PMC8884029

[14] Schmidt A, Ilango SM, McManus MA, Rogers KK, White PH. Outcomes of pediatric to adult health care transition interventions: an updated systematic review. J Pediatr Nurs. 2020;51:92–107. 31981969 10.1016/j.pedn.2020.01.002

[15] Berghetti L, Danielle MBA, Winter VDB, Petersen AGP, Lorenzini E, Kolankiewicz ACB. Transition of care of patients with chronic diseases and its relation with clinical and sociodemographic characteristics. Rev Lat Am Enfermagem. 2023;31:e4014. 37820218 10.1590/1518-8345.6594.4014 PMC10561803

[16] Gray W, Dorriz P, Kim H, Partain L, Benekos E, Carpinelli A, et al. Adult provider perspectives on transition and transfer to adult care: A Multi-Specialty, Multi-Institutional exploration. J Pediatr Nurs. 2021;59:173–80. 33932647 10.1016/j.pedn.2021.04.017

[17] McDonagh JE, Shaw KL, Southwood TR. Growing up and moving on in rheumatology: development and preliminary evaluation of a transitional care programme for a multicentre cohort of adolescents with juvenile idiopathic arthritis. J Child Health Care. 2006;10(1):22–42. 16464931 10.1177/1367493506060203

[18] Sandquist M, Davenport T, Monaco J, Lyon ME. The transition to adulthood for youth living with rare diseases. Child (Basel). 2022;9(5):710. 10.3390/children9050710 PMC9139297 35626888

[19] Pape L, Ernst G. Health care transition from pediatric to adult care: an evidence-based guideline. Eur J Pediatr. 2022;181(5):1951–8. 35084548 10.1007/s00431-022-04385-z PMC9056438

[20] de Las Heras J, Almohalla C, Blasco-Alonso J, Bourbon M, Couce ML, de Castro López MJ et al. Practical recommendations for the diagnosis and management of lysosomal acid lipase deficiency with a focus on Wolman disease. Nutrients. 2024;16(24). 10.3390/nu16244309 PMC11678757 39770929

[21] Torii T, Taniguchi-Fukatsu A, Kawawaki M, Shimoura Y, Ozaki K. Long-Term nutritional counseling for a patient with lipoprotein lipase deficiency. J Atheroscler Thromb. 2023;30(10):1507–15. 36878607 10.5551/jat.63821 PMC10564650

[22] Cleverley K, Rowland E, Bennett K, Jeffs L, Gore D. Identifying core components and indicators of successful transitions from child to adult mental health services: a scoping review. Eur Child Adolesc Psychiatry. 2020;29(2):107–21. 30294756 10.1007/s00787-018-1213-1 PMC7024692

