# Supplementary material for: Childhood to adult transition in youth patients with lysosomal acid lipase deficiency: 43 recommendations from experts
Source: Orphanet J Rare Dis. 2025 Jul 2;20:337. doi: 10.1186/s13023-025-03852-8 (PMC12224453; doi:10.1186/s13023-025-03852-8)
Supplement: Supplementary file 1 — Supplementary Material 1 [file 13023_2025_3852_MOESM1_ESM.docx]

**Supplementary material**

Table S1. Search strategy for each question.

| **Question** | **Search strategy** |
| --- | --- |
| 1-3 | #1. transition pediatric adult care AND challenge AND rare disease  #2. transition pediatric adult care AND barriers AND rare disease  #3. transitioning pediatric adult care AND challenges AND barriers |
| 4 | #1. transition paediatric adult care AND facilitate tools.  #2. transition pediatric adult care AND transition tools.  #3. transition pediatric adult care AND enablers |
| 5 | #1. transition pediatric adult care AND transition plan  #2. transition pediatric adult care AND rare disease AND transition plan  #3. transition pediatric adult care AND rare disease AND transition model |
| 6 | #1. transition pediatric adult care AND transition plan AND age  #2. transition pediatric adult care AND rare disease AND transition plan AND age |
| 7 | #1. transition pediatric adult care AND transition plan AND limitations  #2. transition pediatric adult care AND transition plan AND difficulties |
| 8 | #1. transition pediatric adult care AND transition plan  #2. transition pediatric adult care AND transition plan criteria  #3. transition pediatric adult care AND transition plan characteristics |
| 9 | #1. transition pediatric adult care AND rare disease AND adherence |
| 10 | #1. transition paediatric adult care AND transition plan AND education program  #2. transition paediatric adult care AND transition plan AND patient education |
| 11 | #1. transition paediatric adult care AND transition plan AND education program  #2. transition paediatric adult care AND transition plan AND medical education program |
| 12 | #1. transition paediatric adult care AND transition plan AND education program  #2. transition paediatric adult care AND patient education program |
