# Supplementary material for: Childhood to adult transition in youth patients with lysosomal acid lipase deficiency: 43 recommendations from experts
Source: Orphanet J Rare Dis. 2025 Jul 2;20:337. doi: 10.1186/s13023-025-03852-8 (PMC12224453; doi:10.1186/s13023-025-03852-8)
Supplement: Supplementary file 2 — Supplementary Material 2 [file 13023_2025_3852_MOESM2_ESM.docx]

*Table S2. Publications included in the systematic literature review. The main characteristics (objectives, articles/patients included and conclusions) are indicated.*

| **Reference of the publication** | **Publication type** | **Objective(s)** | **Studies/patients included** | **Conclusions** |
| --- | --- | --- | --- | --- |
| Pérez-López J, et al. Transition process from paediatric to adult care in patients with inborn errors of metabolism. Consensus statement. Med Clin (Barc). 2016;147(11):506.e1-506.e7. | Clinical practice guideline | Establish guidelines to guarantee the success of pediatric to adult care transition | N/A | Crucial role of the internal medicine doctor and the interaction between the pediatric and social setting |
| White PH, et al. Supporting the Health Care Transition From Adolescence to Adulthood in the Medical Home. Pediatrics. 2018;142(5):e20182587. | Clinical practice guideline | Provision of guidance on key elements of the transition process (planning, transfer, and integration into adult care). It also includes the barriers encountered during the process and recommendations | N/A | The health care transition process has an impact on long-term outcomes for young adults  National health surveys should include questions related to the transition process |
| Zubarew T, et al. Transition from pediatric to adult health care services for adolescents with chronic diseases: Recommendations from the Adolescent Branch from Sociedad Chilena de Pediatría. Rev Chil Pediatr. 2017;88(4):553-560. | Clinical practice guideline | Provide recommendations, which support health care professionals involved in the management of adolescents with chronic diseases for the transition process | N/A | Critical challenges encountered during the transition process comprise: the lack of coordination between pediatricians and adult physicians, resistance to transfer or the limited self-management skills development by patients. To overcome these challenges, authors recommend the figure of an accessible professional who coordinates the process and patient education |
| AVEDIS DONABEDIAN. Guía sobre la transición desde los cuidados sanitarios pediátricos a los de adultos  en pacientes con enfermedades crónicas. Instituto Universitario Avedis Donabedian – Universitat Autònoma de Barcelona y Fundación Bancaria “la Caixa”; 2018 [cited 04.09.2024] | Clinical practice guideline | Guideline focused on facilitating the pediatric to adult care transition | N/A | The following points were found to be crucial throughout the transition process: (i) institutional support, (ii) commitment of all health care professionals, (iii) patient and parent education, (iv) figure of the coordinator of a transition process, (v) communication between pediatricians and adult physicians, and (vi) collaboration with patient organizations |
| Willis ER, et al. Transition from children's to adults' services for young people using health or social care services (NICE Guideline NG43). Arch Dis Child Educ Pract Ed. 2018;103(5):253-256. | Clinical practice guideline | Summarize the NICE guideline *Transition from children’s to adults’ services for*  *young people using health or social care*  *services* with a focus on the key points for pediatricians | N/A | The coordination of care is still a challenge in the transition, and specific professionals should be named to facilitate the communication during transitions |
| Antonini TM, et al. Optimization of the transition process of youth with liver disease in adulthood: A position paper from FILFOIE, the French network for paediatric and adult rare liver diseases. Clin Res Hepatol Gastroenterol. 2020;44(2):135-141. | Clinical practice guideline | Recommendations from the Transition Working Group of the French Network for Rare Liver Diseases (FILFOIE) to optimize the transition process of adolescents and young adults with chronic liver diseases | N/A | A successful transition requires: (i) coordination between pediatricians and adult physicians, (ii) independence of young adults, (iii) joint agreement on treatment plans, (iv) preparation on time, (v) education of adult physicians on childhood liver diseases, and (vi) parental support |
| Vajro P, et al. The Health Care Transition of Youth With Liver Disease Into the Adult Health System: Position Paper From ESPGHAN and EASL. J Pediatr Gastroenterol Nutr. 2018;66(6):976-990. | Clinical practice guideline | Propose a pediatric to adult care transition process in patients with pediatric-onset hepatobiliary diseases | N/A | Transition process in these patients is complex and requires the involvement of patients, pediatricians and adult physicians |
| De Castro M, et al. Practical recommendations for the transition to adulthood for the adolescent with  a genetic diagnosis. Special emphasis on inborn errors of metabolism. Transl Sci Rare Dis. 2020;4(3-4):159-168. | Clinical practice guideline | Provide recommendations on the pediatric to adult care transition in patients with inborn errors of metabolism | N/A | To improve patient outcomes during the transition process, provider education is crucial  Genetic counselling for families is recommended |
| Pape L, Ernst G. Health care transition from pediatric to adult care: an evidence-based guideline. Eur J Pediatr. 2022;181(5):1951-1958. | Clinical practice guideline | Guide health care professionals who manage adolescents and young adults with chronic somatic diseases | Inclusion of 40 studies, with 3333 patients aged between 12-28 years | Just a few randomized controlled studies exist on transition, and thus it is not possible to develop evidence-based statements for all areas of the transition process    The standards proposed in this guideline should be implemented so that the national health systems can fund transition process for young adults |
| de Las Heras J, Almohalla C, Blasco-Alonso J, Bourbon M, Couce ML, de Castro López MJ, et al. Practical Recommendations for the Diagnosis and Management of Lysosomal Acid Lipase Deficiency with a Focus on Wolman Disease. Nutrients. 2024;16(24):4309. | Clinical practice guideline | Provide a quick guide for the management of infantile onset LAL-D | N/A | Enzyme replacement therapy and low-lipid diet should be introduced as soon as possible. Genetic counseling and disease awareness programs can play an important role |
| Betz CL, et al. Voices not heard: a systematic review of adolescents' and emerging adults' perspectives of health care transition. Nurs Outlook. 2013;61(5):311-36. | Systematic review | Evaluate health care transition research from the perspective of adolescents and emerging adults with special care needs | Inclusion of 35 studies | More research, which considers adolescents and emerging adults with special health care needs is needed |
| Campbell F, et al. Transition of care for adolescents from paediatric services to adult health services. Cochrane Database Syst Rev. 2016;4(4):CD009794. | Systematic review | Evaluate the effectiveness of the tools used to improve pediatric to adult care transitions | Inclusion of 4 studies (randomized controlled trials) | Due to the limited number of eligible studies, no firm conclusions can be drawn, and further research is needed |
| Doucet S, et al. Programs to support paediatric to adult healthcare transitions for youth with complex care needs and their families: A scoping review. Child Care Health Dev. 2022;48(5):659-692. | Systematic review | Outline the different programs, which exist for children with complex care needs as they transition to adult care | Inclusion of 47 studies | Some recommendations to improve the transition process include the improvement of the coordination, the use of digital tools or the training for health providers |
| Fegran L, et al. Adolescents' and young adults' transition experiences when transferring from paediatric to adult care: a qualitative metasynthesis. Int J Nurs Stud. 2014;51(1):123-35. | Systematic review | Summarize the qualitative studies of pediatric to adult care transition in individuals with chronic diseases | Inclusion of 18 studies | The experiences from young adults seem to be comparable between the different diagnoses. Young adults should be included during the process of transition. Further research in other cultures and health care systems is needed |
| Gabriel P, et al. Outcome Evidence for Structured Pediatric to Adult Health Care Transition Interventions: A Systematic Review. J Pediatr. 2017;188:263-269.e15. | Systematic review | Identify positive outcomes (population health, consumer experience, utilization and costs of care) during the transition from pediatric to adult care | Inclusion of 43 studies | Structured transition often led to positive outcomes |
| Gray WN, et al. Barriers to Transition From Pediatric to Adult Care: A Systematic Review. J Pediatr Psychol. 2018;43(5):488-502. | Systematic review | Identify the barriers during pediatric to adult care transition | Inclusion of 57 studies | Some challenges are shared among the different diseases but some of them are specific for some illnesses. Some barriers included negative beliefs about adult care, difficulty in abandon the relationship with pediatricians, patient or caregiver’s limited knowledge and patient’s lack of self-management skills |
| Marani H, et al. Systematic narrative review of pediatric-to-adult care transition models  for youth with pediatric-onset chronic conditions. Child Youth Serv Rev. 2020;118:105415 | Systematic review | Compare existing models of transition from pediatric to adult care for chronic conditions and identify the common characteristics with positive patient outcomes | Inclusion of 29 studies | There is heterogeneity in the different transition methods applied and inconsistency in the outcomes and quality indicators. However, the incorporation of a facilitator is widely used and presents positive outcomes  Pediatric to adult care transition process for chronic disease requires multiple stakeholders |
| Schmidt A, Ilango SM, McManus MA, Rogers KK, White PH. Outcomes of Pediatric to Adult Health Care Transition Interventions: An Updated Systematic Review. J Pediatr Nurs. 2020;51:92-107. | Systematic review | Assess outcomes of the latest pediatric-to-adult health care transition interventions | Inclusion of 19 studies | A structured transition process improves the management of youth with special health care needs, as it enhances adherence to care, quality of life, self-care skills and satisfaction, among others |
| Stinson J, Kohut SA, Spiegel L, White M, Gill N, Colbourne G, et al. A systematic review of transition readiness and transfer satisfaction measures for adolescents with chronic illness. Int J Adolesc Med Health. 2014;26(2):159-74. | Systematic review | Assess readiness and transfer satisfaction measures for adolescents with chronic illnesses and the psychometric quality of these measures | Inclusion of 14 studies | There is a gap in knowledge of transition care in adolescence with chronic illnesses. Future research is needed to develop well-validated transition readiness questionnaires, validate the existing measures, and reach consensus on outcomes of successful transfer |
| Varty M, Speller-Brown B, Phillips L, Kelly KP. Youths' Experiences of Transition from Pediatric to Adult Care: An Updated Qualitative Metasynthesis. J Pediatr Nurs. 2020;55:201-210. | Systematic review | Update the previous metasynthesis conducted by Fegran, et al (2014) about the experiences and expectations of adolescents and young adults with chronic diseases transitioning to adult care | Inclusion of 33 studies | The findings of this study reaffirmed previous findings. The process of transition should be adolescents and young adults centered and incorporate support for parents |
| Wakimizu R, Sasaki K, Yoshimoto M, Miyazaki A, Saito Y. Multidisciplinary Approach for Adult Patients With Childhood-Onset Chronic Disease Focusing on Promoting Pediatric to Adult Healthcare Transition Interventions: An Updated Systematic Review. Front Pediatr. 2022;10:919865. | Systematic review | Evaluate a multidisciplinary approach toward adult patients with childhood-onset chronic disease, with a focus on pediatric to adult healthcare transition interventions and their effects | Inclusion of 16 studies | The interventions used in the process of transition from pediatric to adult health care provide support for the transition, patient independence, and social participation; and they should be adapted according to the expected effects |
| Bloom SR, Kuhlthau K, Van Cleave J, Knapp AA, Newacheck P, Perrin JM. Health care transition for youth with special health care needs. J Adolesc Health. 2012;51(3):213-9. | Systematic review | Determine adult outcomes for youth with special health care needs and no interventions during the transition, and identify strategies for better transition outcomes to adult health care | Inclusion of 15 studies | Evidence for transition programs is inconclusive. Early introduction to adult providers may improve access to care |
| Sandquist M, Davenport T, Monaco J, Lyon ME. The Transition to Adulthood for Youth Living with Rare Diseases. Children (Basel). 2022;9(5):710. | Study - literature review | Identify barriers and solutions faced by adolescents and young adults with rare diseases and special needs | Inclusion of 14 studies | Transition to adult healthcare is challenging for adolescents with rare diseases. Few of them receive transitional support and racial and ethnic disparities exist.  Cooperation between pediatric and adult physicians is crucial.  More research is needed on transitional care gaps |
| Rutishauser C, Sawyer SM, Ambresin AE. Transition of young people with chronic conditions: a cross-sectional study of patient perceptions before and after transfer from pediatric to adult health care. Eur J Pediatr. 2014;173(8):1067-74. | Cross-sectional study | Compare perceived barriers and the most preferred age for successful pediatric to adult care transition between young adults pre- and post-transfer | 283 pre-transfer and 89 post-transfer young adults | To overcome the transition barriers, a better transition planning with detailed information about the process is crucial |
| Madaleno J, Samyn M, Gonçalves I, de Bruyne R, Kelly D. Current transition management of adolescents and young adults  with liver diseases: a European reference network rare liver  survey (Poster, EASL CONGRESS 2023) | Study - Questionnaire | Explore current practice | 90 responses from 67 centers in 27 countries were collected | The lack of resources is the main limitation in the pediatric to adult care transition process. Standardization of the management of adolescents and young adults and training are required to improve outcomes |
| Moffett M. Transition Readiness Tools to Improve Health Care Transitions  among Adolescents with Inflammatory Bowel Disease (Project) | Study - Project | Prepare adolescents with inflammatory bowel disease for the pediatric to adult care transition | 27 participants from 12-18 years old | Young adults acquire skills on their own even without a transition program. However, this will not facilitate a timely transition to adult care |
| Lestishock L, Nova S, Disabato J. Improving Adolescent and Young Adult Engagement in the Process of Transitioning to Adult Care. J Adolesc Health. 2021;69(3):424-431. | Study - Quality improvement project | Improve engagement of adolescents and young adults and parents/caregivers in healthcare transition. | 1256 adolescents and young adults aged 14-20 years | After the quality improvement project, the engagement of adolescents and young adults and parents/caregivers improved |
| Mulchan SS, Hinderer KA, Walsh J, McCool A, Becker J. Feasibility and use of a transition process planning and communication tool among multiple subspecialties within a pediatric health system. J Spec Pediatr Nurs. 2022;27(1):e12355. | Cross-sectional quality improvement initiative | Develop and implement a tool to facilitate transition planning and communication | 20645 electronic medical records. 89 health professionals completed a survey to assess the tool | Implementation of these tools may improve communication and coordination of care for youth with special health care needs |
| Lemly DC, Weitzman ER, O'Hare K. Advancing healthcare transitions in the medical home: tools for providers, families and adolescents with special healthcare needs. Curr Opin Pediatr. 2013;25(4):439-46. | Study - Review | Review recent national guidelines regarding the transition process and discuss practical tools for pediatricians to implement guidelines | N/A | Although transition planning is crucial for successful healthcare transitions, most pediatricians struggle to achieve healthcare transition goals |
| Bond J, Shanske S, Hoffman R, Ross AM. Piloting a structured developmental tool to assess transition readiness for youth with special health-care needs: A mixed-methods exploration of health-care provider experiences. J Child Health Care. 2020;24(1):92-105. | Multiphase mixed-methods study | Assess transition readiness for youth with special health-care needs and test a structured tool | The study included 28 health-care providers who completed a total of 108 tools | A structured tool can enhance transition readiness assessment, and it can thus optimize the transition process and patient care |
| Kaufmann Rauen K, Sawin KJ, Bartelt T, Waring WP 3rd, Orr M, et al. Transitioning adolescents and young adults with a chronic health condition to adult healthcare - an exemplar program. Rehabil Nurs. 2013;38(2):63-72. | Study, which evaluates a specific transition program for Spina Bifida | Describe the experiences of the Children’s Hospital of Wisconsin Spina Bifida Program Team in developing and implementing a formalized pediatric to adult Spina Bifida Transition Program | N/A | Key recommendations include: stakeholder identification and effective communication, building trust,  collaboration between pediatric and adult providers and evaluation of the components of the transition process |
| Nagra A, McGinnity PM, Davis N, Salmon AP. Implementing transition: Ready Steady Go. Arch Dis Child Educ Pract Ed. 2015;100(6):313-20. | Study - Review | Describe the obstacles found during the implementation of the transition process and report on a successful generic transition programme (‘Ready Steady Go’, UK) | N/A | Effective transition improves long-term outcomes for young adults. Early transition initiation enhances knowledge and skills in young adults. Poorly managed transitions remain a significant issue for many families |
| Mc Govern EM, Maillart E, Bourgninaud M, Manzato E, Guillonnet C, Mochel F, et al. Making a 'JUMP' from paediatric to adult healthcare: A transitional program for young adults with chronic neurological disease. J Neurol Sci. 2018;395:77-83. | Prospective observational study | Describe the JUMP care pathway and evaluate the satisfaction level of parents and patients. JUMP is a program based in Paris that transitions young adults with neurological conditions | Inclusion of 111 patients (median age of 19.7 years) | Satisfaction of patients and their parents with the program was high.  The role of nurse specialists in coordinating the program is likely the key |
| Joshi D, Gupta N, Samyn M, Deheragoda M, Dobbels F, Heneghan MA. The management of childhood liver diseases in adulthood. J Hepatol. 2017;66(3):631-644. | Study - Review | Describe the successful interventions of a transition service | N/A | Adult hepatology training programs should include young adults with liver disease. A multidisciplinary approach is needed to manage young adults with liver diseases.  The transition process should involve young adults and encourage their self-management |
| González F, Roizen M, Rodríguez Celin ML, De Cunto C, Eymann A, Mato R, et al. Validation of the Argentine Spanish version of Transition Readiness Assessment Questionnaire for adolescents with chronic conditions. Arch Argent Pediatr. 2017;115(1):18-27. | Descriptive, cross-sectional, quantitative study | Validate the Argentinian Spanish version of the Transition Readiness Assessment Questionnaire (TRAQ) 5.0 tool directed to adolescents and young adults with chronic conditions | A total of 191 patients were included in the study (mean age of 16.9 years) | The TRAQ 5.0 tool is validated for Argentinian adolescents with chronic conditions |
| Vajro P, Ferrante L, Lenta S, Mandato C, Persico M. Management of adults with paediatric-onset chronic liver disease: strategic issues for transition care. Dig Liver Dis. 2014;46(4):295-301. | Study - Review | Describe the barriers found for adult primary physicians and hepatologists, and discuss the management of these complications in pediatric-onset liver diseases | N/A | Internal medicine residents feel unprepared for chronic childhood-onset illness care, but this is not the case for pediatric residents.  Effective transition programs require integrated efforts from pediatric and adult staff.  Multidisciplinary care is essential for managing these patients.  Nurses can play a pivotal role in the transition process.  Parental involvement is crucial during the transition process.  Adolescents should take a pivotal role in their healthcare |
| Beaudry J, Consigli A, Clark C, Robinson KJ. Getting Ready for Adult Healthcare: Designing a Chatbot to Coach Adolescents with Special Health Needs Through the Transitions of Care. J Pediatr Nurs. 2019;49:85-91. | Pilot study | Test the feasibility of a texting platform aimed at increasing engagement among teenagers while teaching essential self-care skills.  Create educational tools to prepare patients and their families for the transition process | The study included 13 patients aged 14 to 17 | The texting platform increased the engagement among teenagers. Digital formats alone are insufficient for education in transition skills |

*N/A: not applicable*
