# Supplementary material for: Childhood to adult transition in youth patients with lysosomal acid lipase deficiency: 43 recommendations from experts
Source: Orphanet J Rare Dis. 2025 Jul 2;20:337. doi: 10.1186/s13023-025-03852-8 (PMC12224453; doi:10.1186/s13023-025-03852-8)
Supplement: Supplementary file 3 — Supplementary Material 3 [file 13023_2025_3852_MOESM3_ESM.docx]

*Table S3. Check-list proposal.*

| ***LAL-D*** |  | ***CHECK-LIST TRANSITION*** | |
| --- | --- | --- | --- |
| ***Name:*** |  |  |  |
| ***ID:*** | ***Date of birth:*** | | |
|  | ***BASIC*** | ***INTERMEDIATE*** | ***ADVANCED*** |
| ***WHAT IS HAPPENING TO YOU?*** | | | |
| *You understand your disease, the mechanisms that cause it and the organs it affects* | **🗸** | **🗸** | **🗸** |
| *You know your current health status* |  | **🗸** | **🗸** |
| *You are aware of the potential complications* |  | **🗸** | **🗸** |
| *You understands its origin and transmission at the genetic level* |  |  |  |
| ***HOW TO MANAGE YOUR ILLNESS?*** | | | |
| *You understand which lifestyle habits are beneficial and which are harmful* | **🗸** | **🗸** | **🗸** |
| *You are capable of adhering to treatment and attending medical appointments* | **🗸** | **🗸** | **🗸** |
| *You manage the impact of the illness on studies/work* |  | **🗸** | **🗸** |
| *You manage the impact of the illness on social life* |  | **🗸** | **🗸** |
| *You can effectively communicate with the medical team without supervision* |  | **🗸** | **🗸** |
| *You regularly attend appointments alone* |  |  | **🗸** |
| *You have discussed topics related to sexuality and reproduction with your doctor* |  |  | **🗸** |
| *You have discussed issues regarding alcohol and other drugs with your doctor* |  |  | **🗸** |
| *You have discussed mental health topics with your doctor* |  |  | **🗸** |
| *You are familiar with the pediatric and day hospital teams.* |  | **🗸** | **🗸** |
| *You are familiar with the adult and day hospital teams* |  |  | **🗸** |
| *You know your primary care physician* |  |  | **🗸** |
| *You have access to digital health content* |  |  | **🗸** |
| *You can manage a change of appointment* |  | **🗸** | **🗸** |
| *You understand the role of diet in managing LAL-D and the consequences of non-adherence* |  | **🗸** | **🗸** |
| *You are familiar with the enzyme replacement therapy schedule and how to manage missed doses* |  |  | **🗸** |
| ***WHICH FOLLOW-UP DO YOU NEED?*** | | | |
| *You have information about the transition* | **🗸** | **🗸** | **🗸** |
| *You know how to contact the patient organization* |  | **🗸** | **🗸** |
| *You are aware of your treatment plan and appointments* |  | **🗸** | **🗸** |
| *You have a report for the primary care physician and adult team* |  |  | **🗸** |
| *You have a report for travel or for emergencies* |  |  | **🗸** |
